# Quantitative CT Texture Analysis of COVID-19 Hospitalized Patients during 3–24-Month Follow-Up and Correlation with Functional Parameters

**DOI:** 10.3390/diagnostics14050550

**Published:** 2024-03-05

**Authors:** Salvatore Claudio Fanni, Federica Volpi, Leonardo Colligiani, Davide Chimera, Michele Tonerini, Francesco Pistelli, Roberta Pancani, Chiara Airoldi, Brian J. Bartholmai, Dania Cioni, Laura Carrozzi, Emanuele Neri, Annalisa De Liperi, Chiara Romei

**Affiliations:** 1 Department of Translational Research, Academic Radiology, University of Pisa, 56126 Pisa, Italydania.cioni@unipi.it (D.C.);; 2 Pneumology Unit, Pisa University Hospital, 56124 Pisa, Italyfrancesco.pistelli@unipi.it (F.P.); roberta.pancani@ao-pisa.toscana.it (R.P.);; 3 Department of Surgical, Medical, Molecular and Critical Area Pathology, University of Pisa, 56124 Pisa, Italy; m.tonerini@tiscali.it; 4 Department of Translational Medicine, University of Eastern Piemonte, 28100 Novara, Italy; chiara.airoldi@uniupo.it; 5 Division of Radiology, Mayo Clinic, Rochester, MN 55905, USA; 6 2nd Radiology Unit, Department of Diagnostic Imaging, Pisa University-Hospital, Via Paradisa 2, 56124 Pisa, Italy

**Keywords:** lung, CT, COVID-19 pneumonia, follow-up, machine learning, texture analysis, quantitative computed tomography

## Abstract

Background: To quantitatively evaluate CT lung abnormalities in COVID-19 survivors from the acute phase to 24-month follow-up. Quantitative CT features as predictors of abnormalities’ persistence were investigated. Methods: Patients who survived COVID-19 were retrospectively enrolled and underwent a chest CT at baseline (T0) and 3 months (T3) after discharge, with pulmonary function tests (PFTs). Patients with residual CT abnormalities repeated the CT at 12 (T12) and 24 (T24) months after discharge. A machine-learning-based software, CALIPER, calculated the CT percentage of the whole lung of normal parenchyma, ground glass (GG), reticulation (Ret), and vascular-related structures (VRSs). Differences (Δ) were calculated between time points. Receiver operating characteristic (ROC) curve analyses were performed to test the baseline parameters as predictors of functional impairment at T3 and of the persistence of CT abnormalities at T12. Results: The cohort included 128 patients at T0, 133 at T3, 61 at T12, and 34 at T24. The GG medians were 8.44%, 0.14%, 0.13% and 0.12% at T0, T3, T12 and T24. The Ret medians were 2.79% at T0 and 0.14% at the following time points. All Δ significantly differed from 0, except between T12 and T24. The GG and VRSs at T0 achieved AUCs of 0.73 as predictors of functional impairment, and area under the curves (AUCs) of 0.71 and 0.72 for the persistence of CT abnormalities at T12. Conclusions: CALIPER accurately quantified the CT changes up to the 24-month follow-up. Resolution mostly occurred at T3, and Ret persisting at T12 was almost unchanged at T24. The baseline parameters were good predictors of functional impairment at T3 and of abnormalities’ persistence at T12.

## 1. Introduction

Severe acute respiratory syndrome coronavirus-2 (SARS-CoV-2), the cause of coronavirus disease (COVID-19) [1,2,3], belongs to the betacoronavirus genus and presents genetic similarities with severe acute respiratory syndrome (SARS-CoV-1) and Middle Eastern respiratory syndrome (MERS) [4]. These viruses cause similar radiological and pathological changes, including alveolar epithelial damage, exudation into the alveolar space and the apposition of fibrin-rich hyaline membranes [5,6].

Chest computed tomography (CT) has proven to be mostly a supplemental tool for the diagnosis of SARS-CoV-2 as the reverse transcriptase polymerase chain reaction (RT-PCR) assay is more sensitive and specific [7,8]. However, CT may help to detect the presence of persistent pulmonary abnormalities over time [5,9,10,11].

Indeed, the SARS and MERS viruses are known to have long-term consequences, even after 1 year from infection, resulting in impaired pulmonary function and abnormal CT findings [12,13].

SARS-CoV-2 is also known to result in long-term functional abnormalities, including a decrease in pulmonary function, mainly restrictive patterns and reductions in the ability of the lungs to transfer gases, quantitatively assessed by the diffusion capacity of carbon monoxide (DLCO) [12,13]. However, the frequency, extent and persistence of lung parenchymal abnormalities and fibrosis are still unclear [14,15]. Thus, it is important to implement follow-up strategies to quantitatively evaluate the long-term effects of COVID-19 and determine the potential predictors of long-term sequelae.

Artificial intelligence-based image analysis has become increasingly popular in recent years and received a further attention boost during the pandemic [16,17]. In particular, machine learning (ML), a subfield within artificial intelligence, is dedicated to the development of algorithms enabling computers to learn from and render decisions based on labeled and structured data [18,19]. In this setting, CALIPER, an ML-based software utilizing textural features, has been developed to classify and quantify different pathological lung patterns [20].

This observational study aims to quantitatively evaluate the longitudinal changes in the CT lung findings of COVID-19 pneumonia computed using CALIPER software and to compare these quantitative parameters to pulmonary function tests. As a secondary endpoint, the potential role of quantitative CT parameters in predicting pulmonary function test (PFT) impairment and long-term persistence of CT interstitial abnormalities are investigated.

## 2. Materials and Methods

### 2.1. Subjects and Study Design

The institutional review board approved this retrospective observational single-center study, and informed consent was obtained from all the enrolled patients.

All consecutive patients with COVID-19 pneumonia discharged from Pisa University Hospital from March until September 2020 were considered eligible. The inclusion criteria were as follows: having been hospitalized for COVID-19 pneumonia with at least one RT-PCR of the nasopharyngeal swab positive for SARS-CoV-2 RNA, having accepted to participate in the follow-up program and having performed a non-contrast enhanced chest CT 3 months after discharge. The exclusion criteria were severe motion artifacts in the exams leading to a technically inadequate CALIPER analysis and unavailable RT-PCR during hospitalization.

Patients were evaluated at baseline (T0), three (T3), twelve (T12), and twenty-four (T24) months after discharge. The CT scan at T0 was acquired at the time of admission.

At T3, all patients received a non-contrast chest CT, completed a pulmonary evaluation, pulmonary function tests (PFTs) and underwent DLCO study.

The follow-up CTs were compared to the previous radiological exam by a radiologist specialized in chest radiology with 10 years of experience. Based on this qualitative assessment, patients were divided into group 1, with resolution of all or almost all of the baseline COVID-19 pneumonia signs, and group 2, with residual signs of CT abnormalities or worsening.

Based on the radiological evaluation, patients belonging to group 1 proceeded with the follow-up only with physical examination and PFTs, while patients in group 2 repeated the CT at the following time point, regardless of respiratory symptomatology or PFTs results. Patients with residual signs on the CT performed ad T12 were included to perform CT at T24.

**Figure 1:**
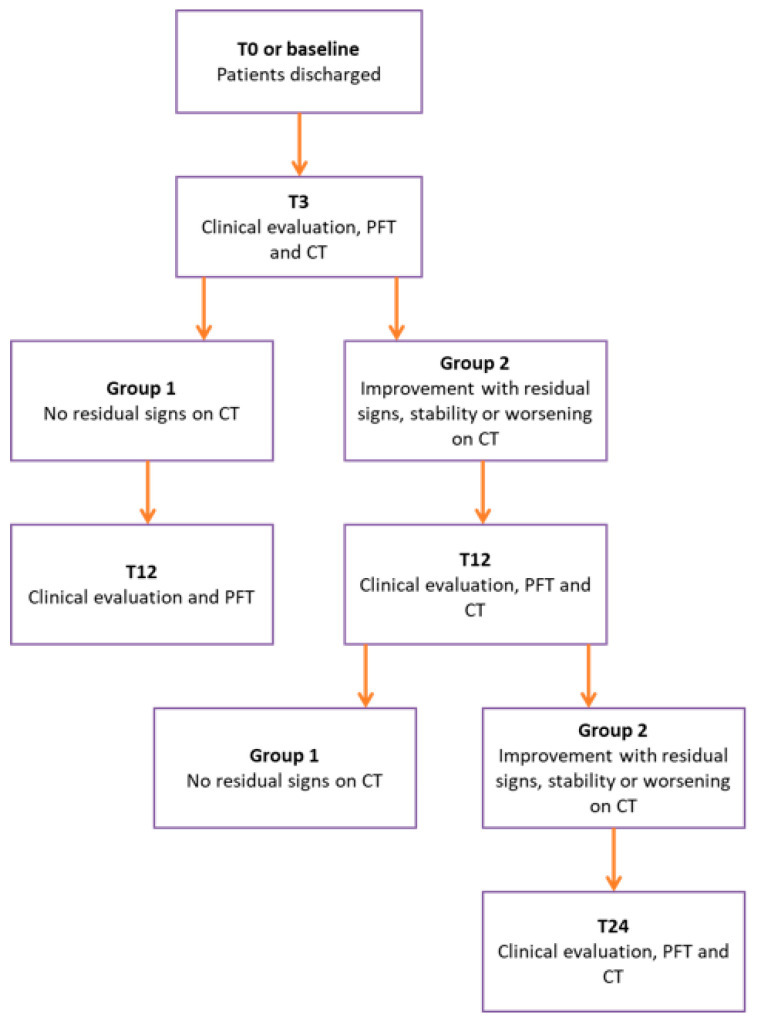
The flow chart adopted for the standardized follow-up of COVID-19 discharged patients.

The follow-up flow chart is presented in Figure 1.

### 2.2. Chest CT Protocols

All the CT examinations were performed throughout the entire lung during a single full inspiratory breath-hold and in a supine position. At T0, two CT scanners were employed to perform all the CTs (64-row General Electric Light Speed and a 40-row Siemens Somatom Sensation scanner).

The General Electric parameters were set as follows: 120 Kv, 169 mAs, 0.98 spiral pitch factor, collimation width 0.625, 512 × 512 matrix, 1.25 mm reconstruction thickness, and standard, soft, and bone plus kernel. The Siemens parameters were set as 120 Kv, 284 mAs, 1.84 spiral pitch factor, collimation width 0.6, 512 × 512 matrix, 1.5 mm reconstruction thickness, and B31 and B70 kernel. The follow-up CTs were acquired using a 64-slice Siemens Somatom Sensation scanner (Siemens Healthineers,, Erlangen, Germany) with the following parameters: 120 Kv, 250 mAs, collimation width 0.6, 512 × 512 matrix, 1 spiral pitch factor, 1.5 mm reconstruction thickness, and B60, B31 and B35 kernel.

### 2.3. CALIPER Texture Analysis

All the CT scans were analyzed with CALIPER, a machine-learning-based texture analysis software trained for the classification of parenchymal features in the setting of interstitial lung disease (ILD) [21]. CALIPER software performs lung segmentation through an adaptive-density-based morphologic approach. Then, the software classifies different radiological patterns: normal lung, ground-glass opacities, reticular abnormalities and honeycombing. All the patterns are quantified in liters and these volumes are then calculated as percentages of the whole lung volume.

The percentage of ILD was defined as the sum of the percentages of GGO, reticular abnormalities and honeycombing [20]. CALIPER allows the computing of the vascular-related structure (VRS) volume, defined as the volume of pulmonary vessels and contiguous-related structures, as perivascular fibrosis [22]. To calculate this parameter, the software utilizes the eigenvalues of the Hessian matrix, with the purpose being to achieve an optimized multiscale filter enhancing tubular structures, such as pulmonary vessels with a diameter larger than 3 mm. The VRS percentage was defined as the ratio of the VRS volume divided by the whole lung volume.

The remaining percentage, not included in the ILD or VRS, was defined as non-involved/normal lung (Norm). The percentages of the above-mentioned parameters will be defined as the GG, Ret, ILD, VRS and Norm. The same kernel analyzed at baseline was chosen for the texture analysis of the follow-up CT.

### 2.4. Pulmonary Function Test

At T3, T12 and T24, the patients were evaluated by PFTs, except in the case of contraindications or inability of the patients to perform the required maneuvers. The following PFT parameters were recorded using a “Vyntus TM BODY” plethysmography (Vyaire Medical Inc. 4.0, Höchberg, Germany) with the software SentrySuite 3.10.6., with corrections for temperature and barometric pressure: forced vital capacity (FVC), total lung capacity (TLC), and DLCO. All the examinations were performed according to the ERS standards [23]. Specific reference equations were applied to express, as percentages of the predicted normal, the observed values of spirometry [24], plethysmography and DLCO [25]. For the purpose of a comparison of the data with previous studies, the PFT parameters were defined as abnormal when below 80% of the predicted value.

### 2.5. Statistical Analysis

Categorical variables were reported using the absolute and percentage frequencies, while for numerical ones, the mean and standard deviation (SD) if normally distributed, or the median with interquartile range (IQR) [25th; 75th percentile] otherwise.

The differences in each CT parameter between time points were calculated and evaluated using parametric or non-parametric tests, as appropriate.

To measure the correlation between the CT and PFT parameters, the Spearman’s rank-order correlation was calculated. The predictivity of the quantitative CT parameters for respiratory impairment and for the persistence of long-term CT abnormalities was evaluated through a logistic regression analysis. ROC curves were obtained, and the AUC and OR were calculated.

*p*-values of less than 0.05 were considered to indicate statistical significance.

Statistical analyses were performed with Stata 15 (StataCorp 2017, College Station, TX, USA).

## 3. Results

The cohort studied included 133 subjects with a mean age of 61.36 ± 13.81 years (range 22–89) and prevalent male sex 84 (63.16%). During the hospitalization, out of 128 patients, 24 (18%) were not oxygenated/ventilated, 70 (52%) required low-flow oxygenation or non-invasive ventilation and 39 (29%) were treated with invasive ventilation in the intensive care unit.

Moreover, 5 patients out of 133 did not perform CT at T0.

**Figure 2:**
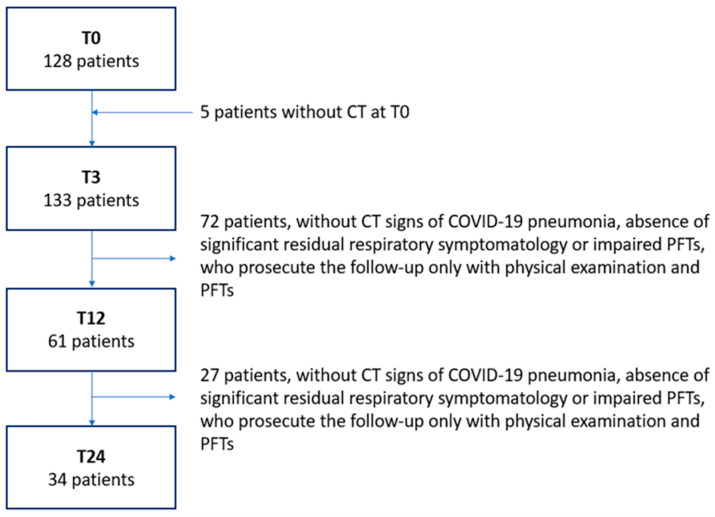
The number of patients for each time point from baseline to T24.

Additionally, 72 patients did not perform the CT at T12, while 61 patients (45.86%) repeated the CT at T12. In this subgroup, 27 patients were followed only with physical examination and PFTs, whereas the remaining 34 patients (25.56%) repeated the CT at T24 (Figure 2).

**Figure 3:**
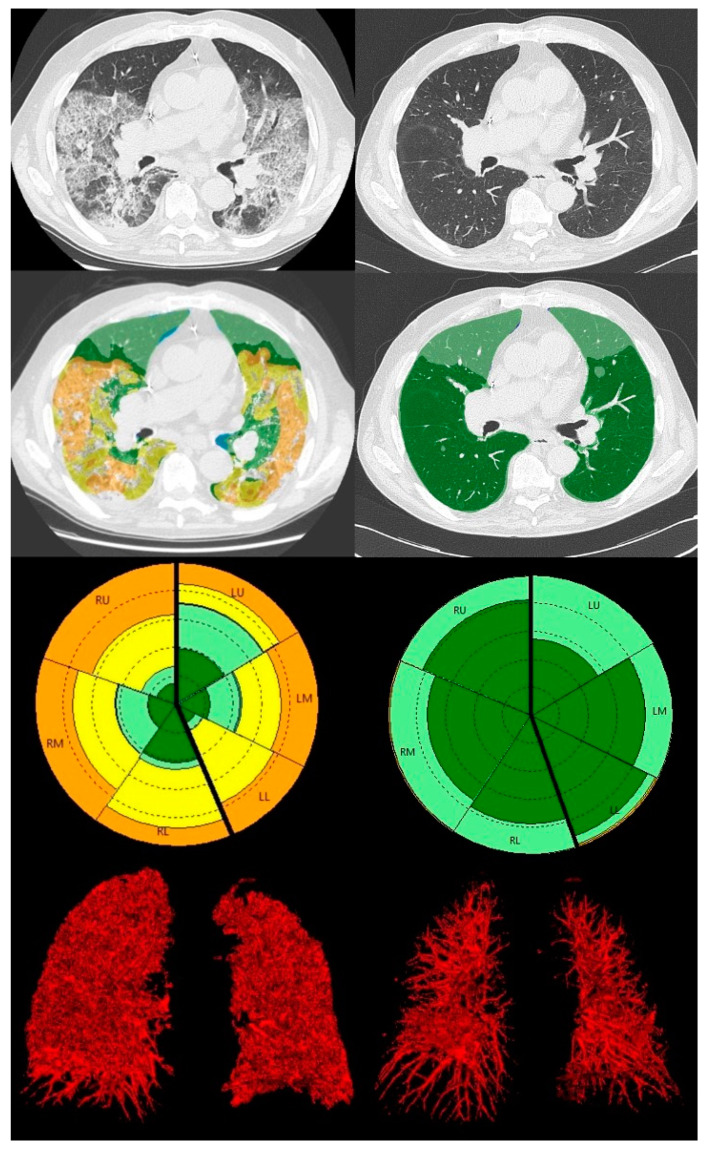
CALIPER analysis of a CT scan of an 84-year-old male patient at T0 (**left**) and T3 (**right**). From the upper to the bottom: axial CT slice, axial CALIPER-derived color image overlays (dark and light green = Norm, yellow = GG, orange Ret), the glyph of the lung parenchymal patterns and the 3D volume rendering reconstructions of lung vessels. Compared to the baseline, after three months, most of the abnormalities are resolved, both GG and Ret. A reduction in the VRS is also demonstrated in the 3D volume rendering.

**Figure 4:**
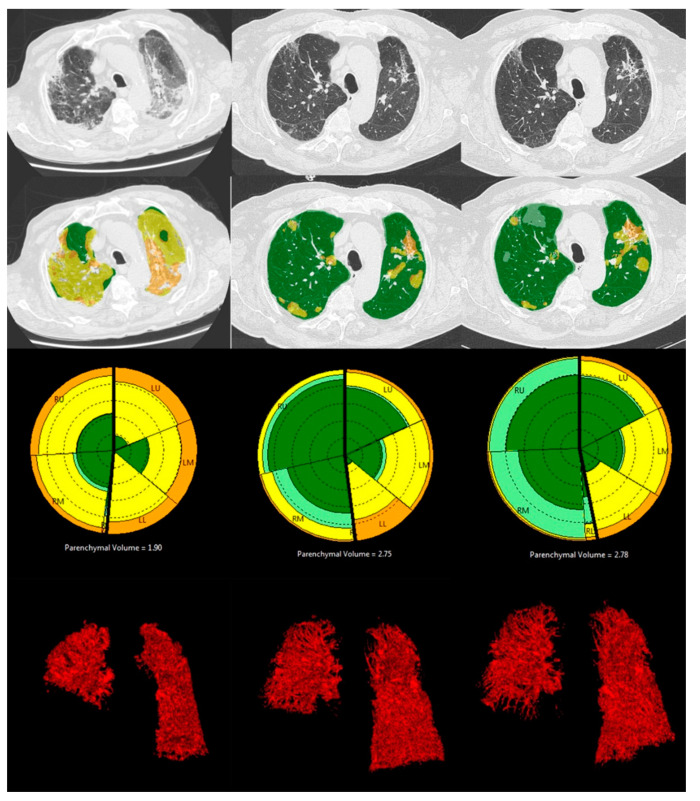
CALIPER analysis of a CT scan of a 92-year-old male patient with right hemidiaphragm elevation and right lower lobe collapse at T0, T12 and T24, from the left to the right. From the upper to the bottom: axial CT slice, axial CALIPER-derived color image overlays (dark and light green = Norm, yellow = GG, orange Ret), the glyph of the lung parenchymal patterns and the 3D volume rendering reconstructions of lung vessels. At T0, the patient presented diffuse areas of GG and Ret. At T12, most of the abnormalities had resolved, while areas of GG associated with Ret persisted, predominantly on the left; these findings persisted also at T24.

The FVC and TLC values were recorded for 121 patients (91%) at T3, 48 patients (78.6%) at T12, and 20 patients at T24 (58.8%). The DLCO was correctly measured for 119 patients (88%) at T3, 45 patients at T12 (73.7%) and 20 patients at T24 (58.8%). The median percentage and interquartile range (IQR) of the CT and PFT parameters for each time point are shown in Table 1 (Figure 3 and Figure 4).

In no patient, either in the acute phase or during follow-up, was honeycombing found, so the ILD should be considered as the sum of the GG and RET.

The delta (Δ) for each CT parameter was calculated at different time points (Table 2).

All the Δ between T3 and T0, between T12 and T0, and between T12 and T3 were significantly different from 0 (*p* < 0.001). All the Δ calculated up to T12 for the Norm showed an increasing trend (Δ > 0), while a decreasing trend (Δ < 0) was observed for all the other parameters. No statistically significant difference from 0 was found for the Δ between T24 and T12.

At T3, 11.58%, 10.84% and 33.90% of subjects had, respectively, FVC, TLC and DLCO ≤ 80% predicted. At T12, the percentages of subjects with functional respiratory impairment were 2.95%, 2.98% and 28.58% for the FVC, TLC and DLCO.

The Spearman’s correlation coefficients between the CT and PFTs parameters at T3 and T12 were calculated. Weak inverse correlations were found between all the quantitative CT and PFT parameters, except for the weak direct correlation between the Norm and PFTs parameters (Table 3 and Table 4). 

**Figure 5:**
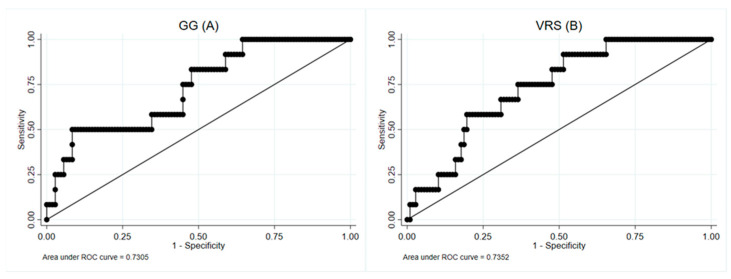
ROC curves for the GG (**A**) and VRS (**B**) measured at T0, considering TLC impairment at T3 as the outcome, with an AUC of 0.73.

Through a logistic regression analysis, the CT parameters were assessed as predictors of PFT impairment at the following time point. When evaluating the CT parameters measured at T0 for the prediction of TLC impairment at T3, the highest AUCs were 0.73, 0.72 and 0.73 for the GG, ILD and VRS, respectively (Figure 5).

**Figure 6:**
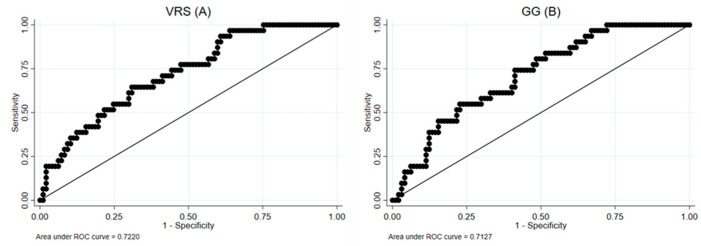
ROC curves for the VRS (**A**) and GG (**B**) at T0, considering the enrollment for CT at T24 as outcome.

A VRS threshold of 3.25% has a sensitivity of 91.67% and a negative predictive value (NPV) of 97.92. No CT parameters at T0 and T3 were predictors of PFT impairment at T12. Similarly, the potential role of the CT parameters at T0 and T3 as predictors was investigated, considering the presence of CT abnormalities at T12 as the outcome. The strongest parameters were the GG and VRS at T0 (AUC, respectively, 0.71, 0.72) and the GG and ILD at T3 (AUC 0.69, 0.70) (Figure 6).

The thresholds were calculated and the optimal cut-offs were 2.51% for the VRS at T0 (sensitivity 93.55%, NPV 94.87%) and 5.25% for the GG at T0 (sensitivity 83.87%, NPV of 90.2%).

## 4. Discussion

This study quantitatively analyzed the longitudinal changes in the CT patterns of COVID-19 pneumonia survivors from baseline to the 24-month follow-up.

As secondary objects, the potential role of quantitative CT parameters in predicting PFT impairments and the persistence of long-term CT lung abnormalities was demonstrated.

The rationale behind a long-term follow-up arises from the need to investigate the possible progression of COVID-19 pneumonia to fibrosis, the radiological stability of this fibrosis over time and the long-term functional consequences [26,27,28].

The CALIPER software was used to quantitatively assess the radiological changes in COVID-19 pneumonia. CALIPER has been utilized in studying interstitial lung disease abnormalities, including its potential as an independent predictor of survival in idiopathic pulmonary fibrosis [21]. Romei et al. first used CALIPER to assess CT scans of COVID-19 pneumonia, and the VRS was demonstrated to be associated with mortality [22].

As previously demonstrated, at baseline, the main lung CT abnormality was GG compared to reticulations [29]. Complete resolution mainly occurred at T3 (65%), similar to that reported by Pan et al. (61%), who performed a visual score of lung abnormalities [15].

In nearly half of our patients, residual abnormalities persisted at T3, mostly GG and to a lesser extent Ret, in line with previous studies. Compared with the qualitative analyses of these studies, our paper precisely quantified these abnormalities, demonstrating very low percentages (<1%) for both the GG and Ret [15,30].

As hypothesized by previous studies, the complete resolution of the GG might be explained by the post-inflammatory change regression, whereas reticulations tend to persist over time [31]. Based on this persistence, 25% of the initial cohort continued the radiological follow-up to T12 and T24, in line with Watanabe et al., who reported a prevalence of 32.6% for patients presenting with residual abnormalities at 1-year of follow-up [32]. At the subsequent follow-up time points, the abnormalities percentage reached a plateau. Indeed, at T24, the Ret percentages were almost completely identical to the T12 ones, and the Δ calculated for the Ret between T12 and T24 was not significantly different from 0. This proportion of reticulations might correspond to the “fibrotic-like changes” described by Martini et al. as potential precursors of fibrosis [10,33,34]. Our findings suggest that this fibrosis/scarring may not ever resolve. However, it is encouraging that the post-COVID-19 pulmonary parenchymal and interstitial processes do not appear to progress as would be expected in inflammatory interstitial lung disease, such as nonspecific interstitial pneumonia, and do not develop progressive traction bronchiectasis or honeycombing as might be seen in idiopathic pulmonary fibrosis.

In SARS-CoV-1 follow-up, lung fibrotic changes’ persistence has been reported by Xie et al. [10,35,36] in 21.5% of patients in a 12-month follow-up, in agreement with the 25% observed in our patients. Zhang et al. followed SARS-CoV-1 survivors up to 15 years and showed the irreversibility of these alterations, with a percentage of pulmonary lesions on CT scans of 4.60 ± 6.37% [10,35,36]. Several reasons could explain the difference with our much lower result (0.31 ILD %). First, our 2-year follow-up is a relatively short period. Second, the quantitative analysis performed by the authors was carried out only on two planes, probably leading to a disease burden overestimation, and they took into account both GGOs and cord-like consolidation [10,35,36].

Regarding the PFTs, in our study, the most common abnormality was the impairment of DLCO, with 33.90% and 28.58% of subjects, respectively, at T3 and T12 presenting with DLCO < 80% predicted. Our results align with those of Torres-Castro et al., who reported a prevalence of 39% for altered DLCO [37].

Additionally, our study analyzed the correlation between the CT parameters and PFTs. Specifically, an inverse correlation trend between the PFT parameters and the percentage of CT abnormalities was found, while there was a direct correlation between the PFTs and Norm. Our results agree with those reported for idiopathic pulmonary fibrosis in previous works, reporting that the CALIPER data of ILD extension were significantly correlated with the PFT [20,38]. However, despite its significance, the weak correlation underscores the nuanced relationship between the CT parameters and PFTs, suggesting potential influences from other factors. Colombi et al. also reported a comparable result in their study, wherein they conducted a visual quantitative assessment of CT scans for COVID-19 pneumonia follow-up. The authors observed a weak correlation between the quantitative parameters and pulmonary function tests (PFTs) [39]. Even more interestingly, the radiological parameters measured at baseline were demonstrated to be predictors of functional impairment at T3. This result may allow the identification in the acute phase of patients who will develop spirometry alteration in the near future. Similarly, Salerno et al. demonstrated that the volume of blood vessels between 5 mm^2^ and 10 mm^2^ in cross-sections measured through deep learning was independently associated with DLCO impairment. It is difficult to make a direct comparison with our work due to the different technique of segmentation and quantification of the vascular volume [40].

The lack of significance when evaluating the radiological parameters at T0 and T3 as predictors of PFT impairment at T12 is probably justified by the longer time interval and the low sample size at T12.

Finally, we assessed the potential role of CALIPER-derived features as predictors of long-term CT interstitial abnormalities at T12. Our results showed that severe patients with higher percentages of GG and VRS during hospitalization have a higher risk of presenting with long-term CT abnormalities. However, with values lower than 0.80, the AUCs may be deemed suboptimal, partly attributable to the relatively small patient cohort and the inherent limitations of the software, which does not account for consolidations. A similar trend is confirmed by Watanabe et al., who reported in their meta-analysis a combined higher proportion for residual CT abnormalities at the 1-year follow-up in severe/critical patients (37.7%) then in mild/moderate patients (20.7%) [32].

For both the logistic regression analyses, thresholds were established, aiming to maximize the ability of the cut-offs to individuate subjects with functional sequelae or long-term CT abnormalities, minimizing the false negatives.

Our study has several limitations and additional efforts are needed to validate our findings. The retrospective nature of this study could account for unforeseen selection bias and confounding factors not taken into account. All the patients enrolled in our study were hospitalized at baseline, thus not reflecting the characteristics of the general population. A monocentric with a relatively small sample size study was conducted, and this is particularly true at T12 and T24, where a large proportion of patients did not repeat the CT, limiting the generalizability of our findings. The lack of spirometry data before COVID-19 pneumonia have to be mentioned, since the attribution of PFT impairment to COVID-19 pneumonia is not totally certain. Finally, the study did not compare the quantitative CT parameters with other clinical or laboratory data.

## 5. Conclusions

CALIPER software allows quantification of lung abnormalities in the setting of acute COVID-19 pneumonia and can be used to track their changes in extent and morphology over time. The CT lung abnormalities not resolving in 3 months, mostly reticular densities, persisted almost unchanged at T12 and even at T24. The CT parameters at baseline were able to predict PFT impairment and the presence of long-term CT abnormalities. Further studies are needed in order to confirm our results.

## Figures and Tables

**Table 1:** Median percentage and IQR values for the quantitative CT and percentage of predicted values for the PFT parameters at each time point.

	T0 (*n* = 128)	T3 (*n* = 133)	T12 (*n*= 61)	T24 (*n*= 34)
GG *	8.44 [3.18; 21.41]	0.14 [0.03; 0.72]	0.13 [0.03; 0.82]	0.12 [0.03; 0.46]
Ret *	2.79 [1.17; 6.77]	0.11 [0.05; 0.50]	0.14 [0.06; 0.49]	0.14 [0.06; 0.44]
ILD *	11.59 [4.99; 28.97]	0.24 [0.1; 1.44]	0.26 [0.12; 1.18]	0.31 [0.12; 0.81]
VRS *	3.65 [2.27; 5.52]	1.72 [1.51; 2.13]	1.75 [1.44; 2.04]	1.72 [1.43; 2.16]
Norm *	84.44 [64.28; 92.59]	97.97 [96.6; 98.31]	97.81 [96.86; 98.29]	97.86 [96.96; 98.25]
FVC **		98 [90; 109]	104 [96; 113]	111.8 [98; 119]
TLC **		96 [88; 106]	100 [93; 107]	99 [92; 106]
DLCO **		90 [77; 99]	88 [77; 99]	87 [74; 98]

* CALIPER parameters are calculated as percentages of the whole lung volume. GG, ground glass. Ret, reticular opacities. ILD, interstitial lung disease, defined as the sum of the GG and Ret. VRS, vascular-related structure. Norm, normal/noninvolved lung. ** Pulmonary function tests are expressed as percentages of the predicted normal. FVC, forced vital capacity. TLC, total lung capacity. DLCO, diffusion capacity of carbon monoxide.

**Table 2:** Δ values (median [IQR]) for each quantitative CT parameter at different time points.

Δ	T3-T0 (*n*= 128)	T12-T0 (*n* = 61)	T12-T3 (*n* = 61)	T24-T12 (*n*= 34)
GG *	−6.58 [−18.92; −2.06]	−13.34 [−25.51; −4.95]	−0.15 [−0.68; 0.01]	−0.01 [−0.08; 0.02]
Ret *	−2.50 [−6.22; −1.02]	−4.34 [−7.1; −1.42]	−0.07 [−0.41; 0.01]	−0.01 [−0.10; 0.04]
ILD *	−10.77 [−25.07; −3.59]	−18.24 [−30.90; −9.91]	−0.18 [−1.01; 0.03]	0.02 [−0.19; 0.06]
VRS *	−1.89 [−3.48; −0.51]	−2.69 [−5.19; −1.62]	−0.13 [−0.39; −0.01]	−0.06 [−0.20; 0.00]
Norm *	12.81 [4.28; 27.49]	21.75 [11.75; 36.11]	0.39 [−0.02; 1.78]	0.04 [−0.08; 0.29]

* CALIPER parameters are calculated as percentages of the whole lung volume. GG, ground glass. Ret, reticular opacities. ILD, interstitial lung disease, defined as the sum of the GG and Ret. VRS, vascular-related structure. Norm, normal/noninvolved lung.

**Table 3:** Correlation between the CT parameters and PFTs at T3.

T3	VRS	Norm	GG	Ret	ILD
TLC	−0.46 *	0.39 *	−0.29 *	−0.27 *	−0.31 *
	>0.00	>0.00	>0.00	>0.00	>0.00
	119	119	119	119	119
FVC	−0.35 *	0.31 *	−0.27 *	0.26 *	−0.31 *
	>0.00	>0.00	>0.00	>0.00	>0.00
	120	120	120	120	120
DLCO	−0.10	0.14	0.06	−0.20 *	−0.14
	0.24	0.12	0.45	0.02	0.12
	118	118	118	118	118

* Statistically significant correlation.

**Table 4:** Correlation between the CT parameters and PFTs at T12.

T12	VRS	Norm	GG	Ret	ILD
TLC	−0.43 *	0.24	−0.20	−0.21	−0.23
	0.002	0.101	0.20	0.14	0.14
	47	47	47	47	47
FVC	−0.36 *	0.21	−0.16	−0.16	−0.22
	0.01	0.13	0.26	0.25	0.13
	48	48	48	48	48
DLCO	−0.32 *	0.31 *	−0.28 *	−0.29 *	−0.30 *
	0.03	0.03	0.05	0.04	0.05
	44	44	44	44	44

* Statistically significant correlation.

## Data Availability

The data presented in this study are unavailable due to privacy restrictions.
